# Identification of Mutations in Distinct Regions of p85 Alpha in Urothelial Cancer

**DOI:** 10.1371/journal.pone.0084411

**Published:** 2013-12-18

**Authors:** Rebecca L. Ross, Julie E. Burns, Claire F. Taylor, Paul Mellor, Deborah H. Anderson, Margaret A. Knowles

**Affiliations:** 1 Section of Experimental Oncology, Leeds Institute of Cancer and Pathology, St James’s University Hospital, Leeds, United Kingdom; 2 Leeds Cancer Research United Kingdom Centre Genomics Facility, Leeds Institute of Cancer and Pathology, St James’s University Hospital, Leeds, United Kingdom; 3 Cancer Research Unit, Saskatchewan Cancer Agency and the University of Saskatchewan, Saskatoon, Canada; University of Central Florida, United States of America

## Abstract

Bladder cancers commonly show genetic aberrations in the phosphatidylinositol 3-kinase signaling pathway. Here we have screened for mutations in *PIK3R1*, which encodes p85α, one of the regulatory subunits of PI3K. Two hundred and sixty-four bladder tumours and 41 bladder tumour cell lines were screened and 18 mutations were detected. Thirteen mutations were in C-terminal domains and are predicted to interfere with the interaction between p85α and p110α. Five mutations were in the BH domain of PIK3R1. This region has been implicated in p110α-independent roles of p85α, such as binding to and altering the activities of PTEN, Rab4 and Rab5. Expression of these mutant BH-p85α forms in mouse embryonic fibroblasts with p85α knockout indicated that all forms, except the truncation mutants, could bind and stabilize p110α but did not increase AKT phosphorylation, suggesting that BH mutations function independently of p110α. In a panel of 44 bladder tumour cell lines, 80% had reduced PIK3R1 mRNA expression relative to normal urothelial cells. This, along with mutation of *PIK3R1*, may alter BH domain functioning. Our findings suggest that mutant forms of p85α may play an oncogenic role in bladder cancer, not only via loss of ability to regulate p110α but also via altered function of the BH domain.

## Introduction

The phosphatidylinositol 3-kinase (PI3K) signaling pathway plays a critical role in the regulation of cell growth, proliferation and survival [1] and mutations that lead to aberrant activation of the pathway are found in virtually all types of cancer. In urothelial carcinoma (UC) of the bladder, several genomic alterations that lead to deregulation of the pathway have been identified. These include inactivating mutations in *PTEN* and *TSC1* and activating mutations in *PIK3CA* and *AKT1* [2,3,4,5,6,7,8]. Previously we showed that several of these events are non-redundant, suggesting that mutation of more than one pathway member may have additive or synergistic effects [4]. These alterations are found both in low-grade, non-invasive and muscle-invasive UC, indicating that this pathway plays a critical role in UC development and suggesting that it represents an important therapeutic target in these cancers.

PI3 kinases phosphorylate the 3` position on the inositol ring of phosphinositol-4,5-bisphosphate (PIP2) to generate the lipid second messenger phosphinositol-3,4,5-triphosphate (PIP3) that activates AKT downstream signaling. The class IA PI3Kαis an obligate heterodimer consisting of the p110αcatalytic subunit (p110α), encoded by the *PIK3CA* gene, and a regulatory subunit, encoded by one of 3 genes, *PIK3R1, PIK3R2* and *PIK3R3*. The regulatory subunits are essential for stability of p110α and in the resting state suppress its catalytic activity [9]. Each has two SH2 domains that can bind activated membrane-bound growth factor receptors or adaptor molecules, altering its conformation, relieving inhibition of p110α and allowing p110α to phosphorylate PIP2 [10].

Mutations of *PIK3R1*, encoding p85α, have been reported in some cancers. In a mouse lymphoma induced by X-irradiation, a truncated (p65) form containing only amino acids 1-571 was identified and shown to confer increased *in vitro* kinase activity on p110α[11]. This truncated form of p85α was later shown to lack the critical inter-SH2 (iSH2) region required for inhibition of p110α activity [12]. A similar truncated form was reported in a human Hodgkin’s lymphoma cell line [13] and 4 smaller deletions, within exons 14 or 15 that encode the iSH2 region, in ovarian and colon cancers [14]. Two splicing mutations were also identified, both of which led to skipping of exon 15. Expression of one of these mutant forms with a deletion of exon 13 resulted in constitutive activation of the PI3K pathway in cells, providing evidence that mutant p85 can act as an oncogene in human cancer. A 9 bp deletion encompassing the exon-intron junction of exon 12 [15] and 9 other mutations, of which 8 were in the iSH2 region were identified in glioblastomas [16] and 15 mutations were found in colon cancer, the majority of which were shown to reduce its p110α-inhibitory activity whilst retaining ability to stabilise the complex [17]. A single *PIK3R1* mutation was recently reported in a study of UC that screened exons 12, 14 and 15, (<1% frequency)[18]. In contrast to these low mutation frequencies, it has recently been reported that 20 - 40% of endometrioid endometrial cancers contain *PIK3R1* mutations, the majority in the nSH2 and iSH2 domains [19,20]. 

Our previous finding of mutations in several components of the PI3K pathway in UC, and the finding of *PIK3R1* mutations in other cancer types, prompted us to search for mutations in *PIK3R1*. As evidence has emerged that that N-terminal domains of p85α have oncogenic functions, we chose to screen the entire coding sequence of *PIK3R1*, rather than focus on exons 12, 14 and 15. Here we report a series of mutations in UC-derived cell lines and primary UC tumours, including deletions in the iSH2 region and a series of missense mutations, several in the breakpoint cluster region homology (BH) domain, which has GTPase activity towards Rab5 [21] and can bind PTEN [22,23]. Our findings suggest that mutant forms of p85α may play an oncogenic role in bladder cancer, not only via loss of ability to regulate p110α but also via altered function of the BH domain. 

## Materials and Methods

### Ethics statement

The study was approved by the Leeds East Research Ethics Committee (99/156) and written informed consent was obtained from all patients.

### Patient Samples and DNA Isolation

Cold cup biopsies of 264 urothelial carcinomas (UC) were collected, snap-frozen and stored in liquid nitrogen. The remainder of the tissue was embedded in paraffin for diagnostic assessment. The tumour panel consisted of 10 pTaG1, 42 pTaG2, 24 pTaG3, 7 pT1G1, 27 pT1G2, 57 pT1G3, 2 pT2G1, 13 pT2G2, 54 >pT2G3, 5 G1, 8 G2 and 4 G3 tumours with no underlying stroma (pTx) and 11 tumours with no information [24,25]. All were transitional cell carcinoma. DNA was extracted from frozen sections and venous blood samples as described previously [4].

### Urothelial cell lines

Forty-four UC cell lines (253J, 5637, 639V, 647V, 92-1, 94-10, 96-1, 97-1, 97-18, 97-24, 97-7, BC-3C, BFTC905, BFTC909, CAL29, DSH1, HT1197, HT1376, J82, JMSU1, JO’N, KU-19-19, LUCC1, LUCC2, LUCC3, LUCC4, LUCC5, LUCC6, LUCC7, LUCC8, MGH-U3, RT112, RT4, SCaBER, SD, SW780, SW1710, T24, TCCSUP, U-BLC1, UM-UC3, VM-CUB-1, VM-CUB-2 and VM-CUB-3) were investigated (Table S1). LUCC1-LUCC8 lines were established in the authors’ laboratory from bladder tumour tissues obtained with written informed consent. Cell line identity was verified by short tandem repeat DNA typing using Powerplex 16 kit (Promega). Profiles were compared to publically available data (ATCC, DSMZ) or where no reference profile was available, were confirmed as unique. DNA was extracted as previously described [4].

### Mutation analysis

 The entire coding sequence of *PIK3R1* was examined in 18 fragments by high resolution melting analysis as described [26,27]. DNA from matched blood samples was analyzed to confirm somatic mutation status. Primer sequences are given in Table S2. Mutations were recorded with reference to NM_181523. 

 Allele-specific PCR (AS-PCR) was carried out to establish the phase of the pairs of mutations in the cell line LUCC3 and in tumour sample 2 (Table 1). A forward primer was designed to specifically amplify the mutant allele at the 5` mutation site and was matched with a reverse primer positioned to include the second mutation in the PCR product (Table S3). Products were run on an agarose gel to identify successful amplification from tumour/cell line DNA only and no amplification from blood and WT DNA. PCR products were sequence-verified.

**Table 1:** Mutations in PIK3R1 identified in bladder tumours and cell lines.

**Sample**	**Grade/stage**	**Exon**	**Nucleotide change**	**Predicted amino acid change**
1	TaG3	2	^1^c..409G>A	E137K
2	TxG3	5	c.652GT	E218*
		6	c.862AC	K288Q
		intron 12	c.1426-49 G>A	unknown
3	T4G3	5	c.710_727del18	W237-Y242del
4	T1G3	5	c.785GC	R262T
5	TaG3	10	^2^c.1072CT	R358*
		14	c.1735_1740del6	Q579-Y580del
6	T1G3	11	c.1131TG	N377K
7	TaG3	12	c.1322AT	N441I
8	T1G1	13	c.1441AT	R481W
^3^LUCC3	T2G3	13	c.1519GC	E507Q
		14	c.1670GC	R557P
9	T2G3	13	c.1552GC	E518Q
10	T1G3	14	c.1585GA	D529N
11	TaG2	14	c.1696_1734del39	I566-D578del
12	T1G2	16	c.1934CT	T645I
13	TaG2	Exon 17 splice acceptor	c.1986-1 G>C	unknown

^1^cDNA reference sequence: NM_181523; ^2^ mutation in only a minor population of sequences; ^3^cell line.

### Expression vectors and transduction of cell lines

Cloning of inserts encoding the full-length wild-type (WT) human p85α and bovine p85α R274A mutant into pGEX-6P (GE Healthcare Life Sciences) has been described [21]. Mutants E137K, R162*, E218*, Δ237_242, R262T, K288Q were created by site-directed mutagenesis using the QuikChange method (Stratagene, CA, USA) and cloned into pFB Hyg [28] in-frame with an N-terminal HA tag using the InFusion method (Clontech, CA, USA).

Control cDNAs, N564D and p85Δ, were kindly provided by Genentech [17] and subcloned into pFB Hyg by the same method. All plasmids were sequence-verified. Constructs were transfected into Phoenix A cells using TransIT 293 (Mirus, Madison, USA). Mouse embryonic fibroblasts (MEFs) with knockout of p85α, β, δ(a kind gift from Lewis Cantley, Harvard Medical School), NIH3T3 mouse fibroblasts and Rat1 rat fibroblasts, were incubated with retroviral supernatant containing 8 μg/ml polybrene and selected with 500, 200 and 250 μg/ml hygromycin, respectively.

### Functional characterization of BH domain mutants

Cells were lysed and protein extracted using CelLytic Mammalian Cell Lysis Reagent (Sigma-Aldrich, Dorset, UK) according to the manufacturers’ instructions and quantified as previously described [29]. Anti-HA Immunoprecipitation Kit (Sigma-Aldrich) was used according to the manufacturers’ instructions for co-immunoprecipation experiments. Immunoblotted proteins were incubated with primary antibodies; anti-p85α (Abcam, Cambridge, UK), anti-p110α, anti-pAKT (Ser473), anti-panAKT (Cell Signaling Technology, Boston, UK) and anti-tubulin alpha (AbD Serotec, Kidlington, Oxford, UK). Bound primary antibodies were detected using HRP-conjugated secondary antibodies and Luminata Forte Western HRP Substrate (Millipore, Watford, UK). Anchorage-independent growth was carried out as previously described [29] and colonies with a diameter ≥50 μm were counted within 2.5 mm^2^.

### Analysis of PIK3R1 mRNA and protein expression in UC

PIK3R1 mRNA expression data for 105 bladder tumour samples and 52 normal bladder samples reported by Sanchez-Carbayo et al [30] were accessed. PIK3R1 protein expression was assessed in 44 UC cell lines and pooled normal human urothelial cells (NHU-pool) by western blotting and normalized to tubulin.

## Results

### Identification of somatic *PIK3R1* mutations

**Figure 1:**
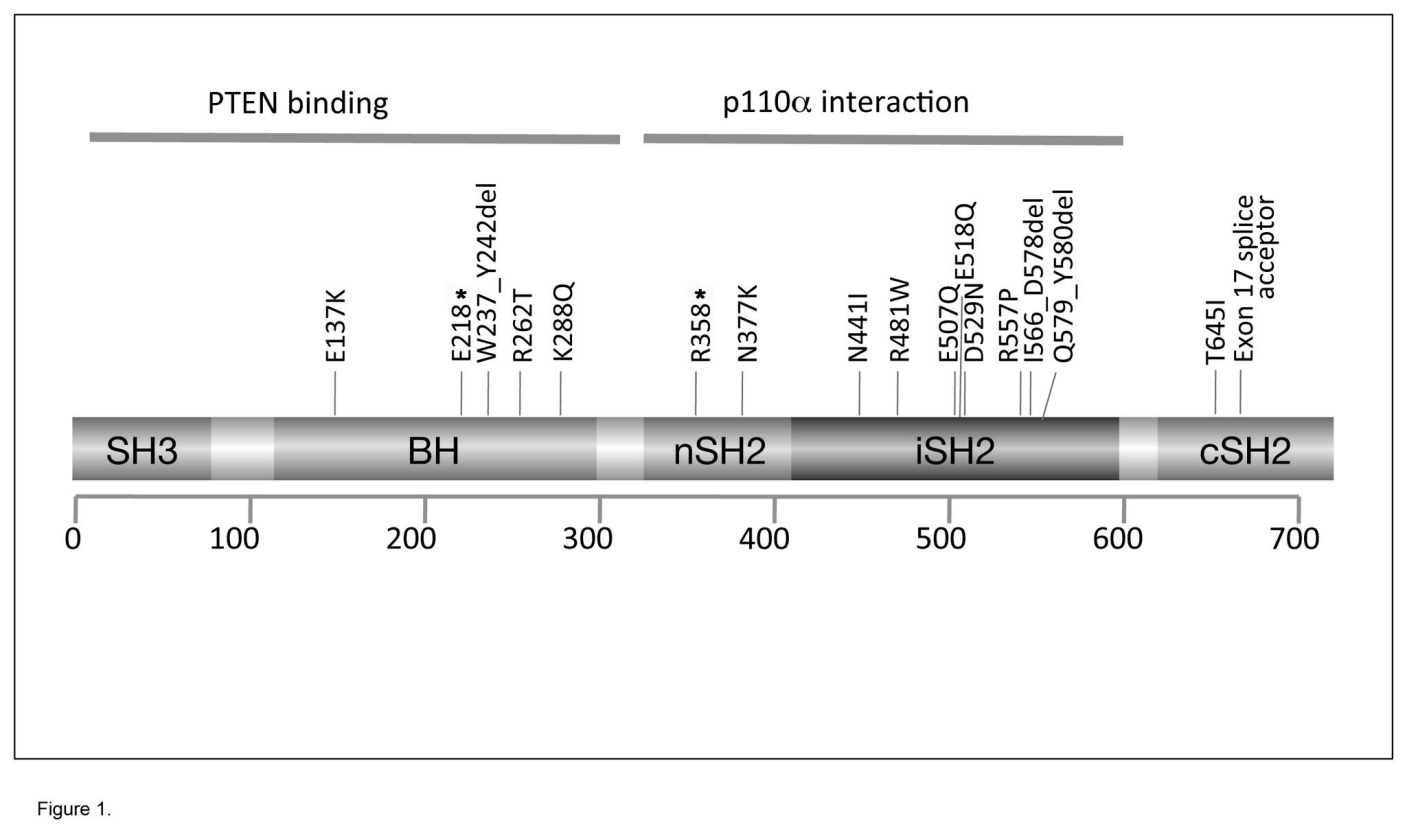
Distribution of *PIK3R1* mutations identified in UC.

We screened the entire coding sequence of *PIK3R1* for mutations in 264 UC tumour tissues and 41 UC cell lines. The three known isoforms of PIK3R1, p85α p55α and p50α contain different exons. Exons 1-6 are unique to p85α, exon 7 is present only in p50α and exon 8 only in p55α. Exons 9-17 are present in all isoforms but the use of a cryptic splice site within exon 16 results in the generation of cDNAs that differ in length by 8 codons [31], both of which were covered by this screen. Eighteen mutations were identified in 13 tumours. One tumour (tumour 2) contained three and one (tumour 5) contained two mutations (Table 1). All were confirmed as somatic mutations by their presence in the tumour-derived DNA but not the patient’s blood. One tumour-derived cell line (LUCC3) contained two mutations and these were also confirmed as somatic in origin. All mutations were confirmed by repeat PCR amplifications to rule out PCR artifacts. The nature and distribution of the mutations are shown in relation to p85α protein structure in Figure 1. Five were located in the BH domain (Figure S1). None were in exons that are unique to p50α or p55α, though several were located in exons 2, 5 and 6, that are unique to p85α. No mutations were identified in the region of exon 16 that is alternatively spliced. 

 Allele-specific PCR (AS-PCR) was used to determine whether the 2 mutations in the cell line LUCC3 (c.1519GC and c.1670GC; E507Q and R557P) and the 2 exonic mutations in tumour 2 (c.652GT and c.862AC; E218* and K288Q) were present in *cis* or in *trans*. In LUCC3, a mutation-specific primer for c.1519C successfully amplified a PCR product from exon 13 to 14 (Figure S2A). Sequencing of this product confirmed the presence of mutation c.1670C, indicating that both iSH2 domain mutations are on the same allele. AS-PCR analysis of tumour 2 also confirmed that the 2 exonic mutations (BH domain E218* and K288Q mutations) were present on the same allele (Figure S2B). 

In tumour 5, sequence profiles indicated that one of the mutations (R358*) was present as only a minor population of molecules, whilst the other (Q579_Y580del) appeared heterozygous. The genomic distance between exons 10 and 14 precluded development of an allele-specific PCR assay to determine whether these mutations were on the same allele. The small signal from the R358* mutation may indicate that it was present as only a minor sub-clonal event. This appeared to be the case, as analysis of tissue from a subsequent disease recurrence in this patient showed only Q579_Y580del.

### Predicted Effect of Mutations on Protein Function

**Figure 2:**
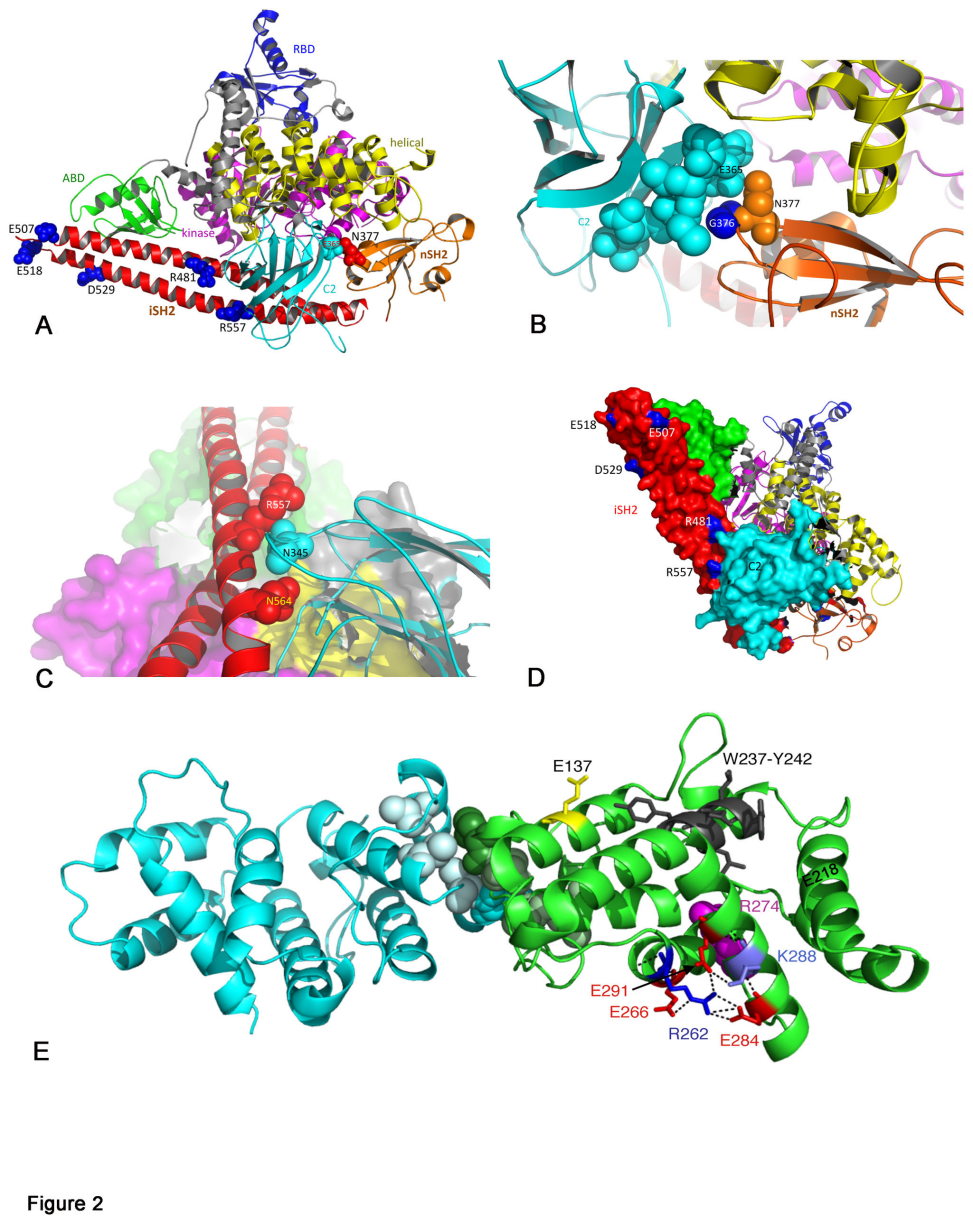
Position of mutations in the p85α protein. A. Ribbon diagram of the structure of the complex of p110α with the niSH2 region of p85α (PDB code 2RD0) showing residues mutated in UC. B. Relationship of p85α nSH2 to p110α C2 domain showing proximity of N377 in nSH2 to C2 domain residue E365. C. Relationship of the iSH2 domain of p85α with the C2 domain of p110α showing proximity of R557 to N345 in C2. D. Space-filling model showing R481 and R557 residues in iSH2 of p85α in contact with C2 of p110α. E. Structure of p85 dimer (PDB code 1PBW) showing position of PIK3R1 point-mutated residues E137, R262 and K288 and the region deleted (W237-Y242) in UC in relation to residues involved in p85 dimerization (M176, dark green/light cyan; L161, I177 and V181, light green/cyan). The position of residue R274 (magenta), which is implicated in Rab-GAP activity is also shown. In addition, three glutamic acid residues (E266, E284 and E291) that interact with R262 are indicated, with E291 also interacting with K288.

The positions of the missense mutated residues that are represented in the available crystal structure are shown in Figure 2. Two mutations, R358* and N377K, were identified in the nSH2 domain. Recent findings suggest that this region of p85α acts as a scaffold for the p110α - p85α complex, with interactions with C2, helical and kinase domains of p110α and with p85α iSH2 [32]. R358* truncates the protein at the nSH2 phosphopeptide binding site (FLVRDAS). This same mutation was recently reported in 5 endometrial cancer samples where it represented 5% of all mutations identified [20]. Arginine 358 has been identified as forming a salt bridge with E542 in p110α [32] and the engineered mutation R358A was shown to abolish phosphopeptide binding [9]. Thus, loss of R358 and truncation at this point is likely to result in disruption of phosphopeptide binding and interaction of p85α with the helical domain of p110α and may mimic the effect of p110α helical domain mutations. We have shown previously that the helical domain mutations E542K and E545K are by far the most common mutations found in *PIK3CA* in bladder cancer [4] and are more potent than H1047R in inducing signaling downstream of AKT and proliferation at confluence and under conditions of nutrient depletion when expressed in normal urothelial cells [29]. As the R358X truncation mutant removes both iSH2 and cSH2 regions of the protein, interaction with the C2 domain of p110α is lost and effects may mimic those described for mutations and truncations affecting these domains [33]. From sequencing data, and knowledge that there was minimal contaminating normal tissue in the sample, this mutation appeared to be heterozygous. Thus it is predicted that in addition to the truncated protein, non-mutant protein was available for p110α binding and stabilisation in this tumour. 

 N377, which was mutated to lysine, is adjacent to G376, which was found previously to be mutated to arginine in 3 cases of glioblastoma [16,34]. Both residues are within a region (374-377) that is predicted to interact with residues 364-371 in the C2 domain of p110α [32]. In the crystal structure of p110α in complex with p85niSH2, both G376 and N377 are within hydrogen bonding distance of E365 in p110α C Figure 2B. 

 Six missense mutations and two in-frame deletions were identified in the iSH2 domain. This is the region where the majority of mutations have been found in other cancers, though none of the alterations found here are identical to those reported previously [14,16,17,19,20]. This region of the protein is involved in interaction with the adapter-binding region of p110α and in stabilisation and inhibition of its catalytic activity in the basal state via interaction with the C2 domain. The predicted effect of some mutations in iSH2 is to disrupt the interface with p110α C2 [16,33]. For example, p85α residue, N564, is within hydrogen-bonding distance of N345 in p110α C2 (Figure 2C), a residue that has been found mutated in p110α [35]. D560Y reported in glioblastoma [16] is also within hydrogen bonding distance of N345 and both of these are predicted therefore to mimic the oncogenic N345 mutation. Here we found a novel mutation, R557P that is also close to N345 of p110α (Figure 2C). R481, which was found mutated to tryptophan, is also in close proximity to the C2 domain of p110α Figure 2D).

 The two in-frame deletions identified in iSH2 (I566_D578del and Q579_Y580del) remove amino acid residues that have been found to be deleted in ovarian and colorectal cancers [14,17] and glioblastoma [16] and are predicted to disrupt the helical secondary structure in this region and disturb interaction with p110α C2. The mutation Q579_Y580del has been reported to bind and stabilise p110α but have reduced ability to regulate the lipid kinase activity of the p85α p110α holoenzyme [17]. Recently, nine of the nSH2 and iSH2 mutations found in other cancers were expressed in chicken embryo fibroblasts and all induced transformation, measured by focus-forming activity, increased proliferation and enhanced signaling via PI3K [36]. Specific inhibitors of p110α, but not inhibitors of p110β p110γ, or p110δ abolished the phenotypic effects of two of these tumour-derived mutant forms (KS459delN, DKRMNS560del), indicating that the oncogenic activity of these mutants is uniquely mediated by p110α.

 The most N-terminal iSH2 mutation identified, N441I, is in a linker region between nSH2 and iSH2, and lies close to the end of the second alpha helix of iSH2. This region is not resolved in the published crystal structures and its potential effect is not clear. T654I in the cSH2 domain is close to several residues that are mutated in colorectal cancers [17] and is immediately adjacent to the FLVRES motif (residues 646-651) required for phosphotyrosine engagement. A serine or threonine residue in this position is found in the SH2 domains of a wide range of proteins. Several mutations within this domain have been reported in colorectal cancers [17] but not in glioblastoma [16,34], raising the possibility that there may be tissue specific differences.

Interestingly, we found five mutations within the BH domain of PIK3R1 (28% of mutations found) (Figure 1; Figure S1). Previous mutation screens have identified only seven mutations in this region (K142fs, E160*, R162*, I177N, E217K, N285H, E297K) in more than 1550 samples of other tumours reported to date [14,16,17,18,19,20,34] (< 0.5%). All missense mutations in this region (E137K, R262T and K288Q) are in highly conserved residues (Figure S3). The structure of this region of the protein (amino acids 105-319) as a homodimer has been reported [37]. The interface between BH domains was shown to involve interaction of M176 from one monomer with L161, I177 and V181 on the other. The three point mutations and deletion identified here are remote from this region of interaction (Figure 2E).

### P85α BH domain mutants interact with and stabilise p110α

To determine whether BH domain mutant proteins have p110α dependent effects, we tested their ability to interact with and stabilise p110α. p85α, β δ knockout (pan-p85 null) MEFs were transduced with retroviral expression vectors encoding N-terminus HA-tagged p85α cDNA encoding the BH domain mutant forms identified in UC (E137K, E218*, Δ237_242, R262T, K288Q), other mutant forms as controls (R162*, p85Δ, R274A-bovine, N564D), wild-type (WT) or empty vector. R162* mutant p85α is a BH domain mutation previously identified in cancer [17] and like p85Δ lacks the p110α binding region (n-iSH2) [38]. Both have previously been shown not to interact with p110α [17]. N564D iSH2 mutant p85α was used as a positive control as it has previously been shown to bind p110α, increase PI3K and AKT activity, and induce cell survival, anchorage-independent growth and transformation-related phenotypes that were p110-dependent [17]. It was also used here to assess potential differences between BH and iSH2 domain mutant forms of p85. R274A is an engineered BH domain mutant form of bovine p85α that has been previously shown to inhibit p85-GAP activity and was oncogenic [21,39].

**Figure 3:**
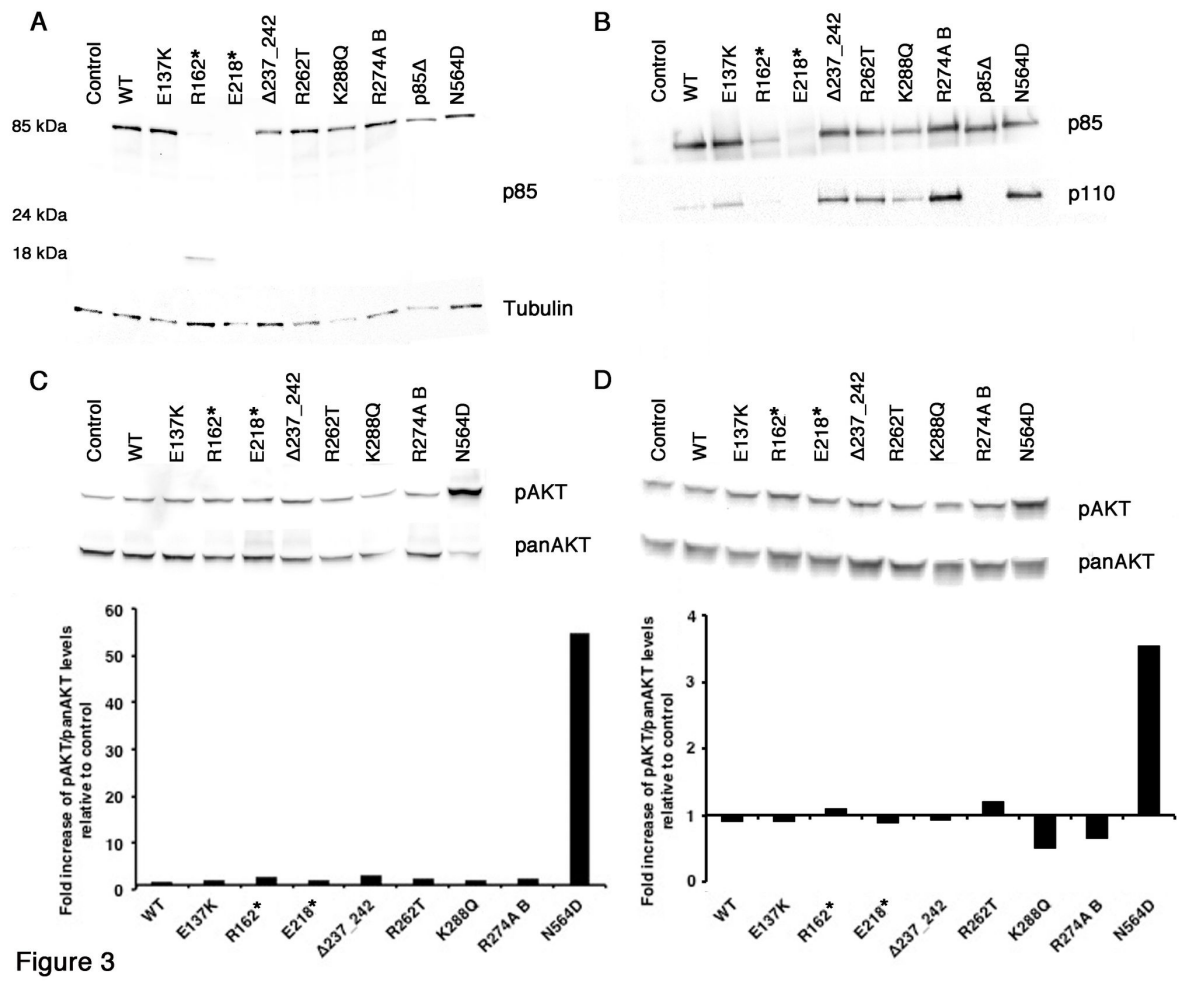
Characterization of MEFs (KO p85α, β, δ) expressing WT or mutant p85α. A. Immunoblot showing p85α protein expression levels in transduced cells. B. Co-immunoprecipitation of HA-tagged proteins followed by immunoblot with p85α and p110α to determine p85-p110 binding. C. Immunoblot analysis of PI3K signaling (levels of pAKT (Ser473)) in serum-containing medium and bar chart displays the quantification of normalized pAKT to total AKT relative to control. D. Immunoblot analysis of pAKT (Ser473) in serum-containing medium and bar chart displays the quantification of normalized pAKT to total AKT relative to control.

Expression of p85α was confirmed by western blotting (Figure 3A). A truncated p85α protein was visible in the R162*-MEF (18 kDa), but in the E218*-MEF, the truncated protein (24 kDa) was expressed at a very low level. Three independent transductions with E218* virus consistently induced low protein expression suggesting that this protein may be unstable. Interestingly, R162*-MEF also expressed a small amount of full-length p85α protein. 

Pan-p85 null MEFs have low levels of p110α, which can be restored by expression of wild-type p85α [40]. We confirmed this and showed that all mutant forms except E218*, R162* and p85Δ had the same effect (data not shown). Co-immunopreciptation of HA-tagged p85 proteins was used to assess p110α binding (Figure 3B). Results indicated that all p85 proteins, except E218*, R162* and p85Δ, could bind p110α. 

### BH and iSH2 domain mutants of p85α show different effects on AKT activation and anchorage-independent growth

p85α C-terminal domain mutant forms that affect p110α activity normally result in activation of AKT. This has been demonstrated for several p85α mutants that can bind p110α [17,19,20]. In pan-p85 null MEFs reconstituted with wild-type and mutant forms of p85α, we found that only the N564D mutant induced increased levels of phospho-AKT (Ser473) both in serum-containing medium (Figure 3C), and in conditions of serum-deprivation (data not shown). This was also seen in NIH3T3 expressing wild-type and mutant forms of p85α when maintained in serum-supplemented conditions (Figure 3D). This is consistent with the previous finding in NIH3T3 that bovine R274A does not affect phosphorylation of AKT following stimulation with PDGF [21]. This pattern was reflected in the proliferation rates of MEF and NIH3T3 cells when maintained in serum-containing medium and when assessing anchorage-independent growth of NIH3T3 cells (data not shown). Analysis of anchorage-independent growth of Rat1 cells expressing wild-type and mutant forms of p85α showed that whilst BH domain mutants induced some colony formation relative to wild-type p85α, N564D had the greatest effect (Figure S4).

### Relationship to other mutations in PI3K pathway genes

Previously we screened the samples analyzed here for *PIK3CA* mutation [[4] and unpublished data]. Only two samples contained both *PIK3R1* and *PIK3CA* (E545K) mutations and one of these was the tumour that had a BH domain mutation (E137K) in *PIK3R1*. Overall, 61 of the tumours screened contain *PIK3CA* mutations (23%). Thus, *PIK3CA* mutation was underrepresented in the subset that had *PIK3R1* mutation (1 observed; 5 expected). Thus *PIK3R1* mutations not in the BH domain appear to be mutually exclusive with *PIK3CA* mutation in UC. There was no significant relationship of *PIK3CA* or *PIK3R1* mutation with tumour grade or stage. Four of the samples contain *AKT1* mutations (E17K), none of which co-existed with *PIK3R1* mutation. *TSC1* mutation status is known for 148 tumours in the series, of which 14 contain a mutation [4]. One of these tumours had a mutation in the BH domain of *PIK3R1* (W237_Y242del). However the sample size and mutation frequencies are too small for any significant coincidence or mutual exclusivity to be identified. 

### PIK3R1 expression is reduced in UC and cell lines

As p85α mutations may alter protein:protein interactions of p85α, some of which, particularly those in the BH domain, might indicate a tumour suppressor role, we considered the possibility that downregulation of the protein might contribute to tumour development.

**Figure 4:**
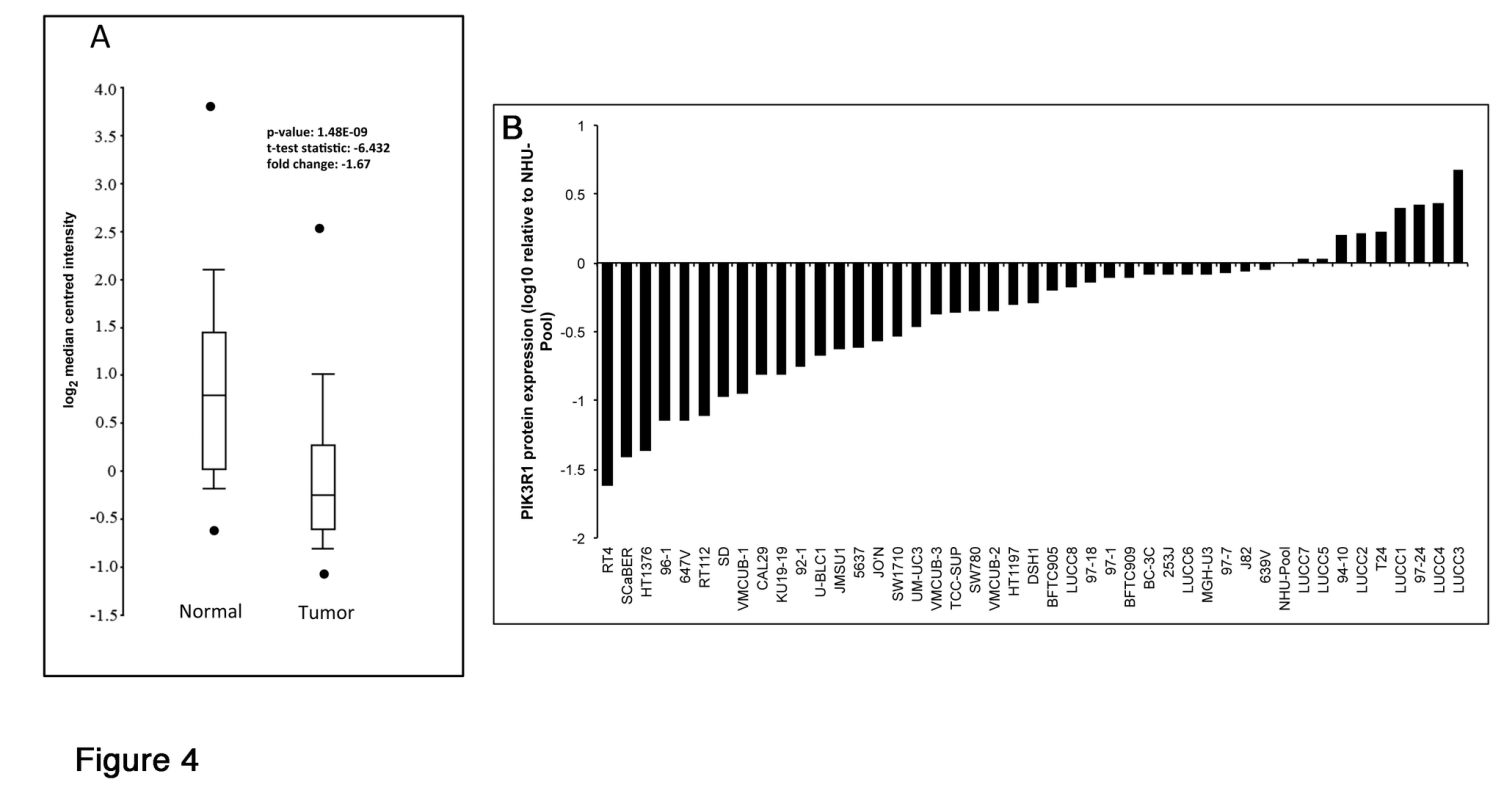
Expression of p85α in bladder tumours and cell lines. A. Analysis of p85α mRNA expression levels in 105 bladder tumour samples and 52 normal bladder samples. Data from Sanchez-Carbayo et al [30]. B. Quantitative analysis of p85α immunoblotted protein samples from bladder cancer cell lines normalized to tubulin and shown relative to pooled normal human urothelial cells (NHU-pool).

*PIK3R1* is located on chromosome 5 (5q13.1). Loss of heterozygosity (LOH) and copy number loss of sequences on 5q have been reported in bladder cancer [41,42]. By array CGH we have found that 29% of muscle invasive UC have copy number loss in the region of *PIK3R1* [43]. Examination of publically available expression data indicated that UC has a significant reduction in PIK3R1 expression at the RNA level (Figure 4A). 

We assessed p85α protein expression in 44 UC cell lines using western blotting. This revealed wide variation in expression levels with the majority of cell lines (80%) showing reduced p85α protein expression compared with normal urothelial cells (Figure 4B).

## Discussion

In this study we found *PIK3R1* mutations in 4.9% of UC. The higher frequency found here than in the previous study of UC [18] reflects our assessment of the entire gene rather than only exons 12, 14 and 15. Our data for those three exons revealed 5 mutations (1.8% of tumours), compatible with this earlier study. The majority of mutations were located in the C-terminal region of p85α similar to findings in other human cancers [14,16,17,19,20]. Mutations identified in the nSH2 and iSH2 domains may mimic the effect of p110α mutations by relieving the inhibitory regulation of p110α by p85α and the oncogenic activity of these mutants appears to be p110α dependent [36].

Interestingly, we found a higher frequency of BH domain mutations compared to previous mutation screens. Whether this is specific to UC is not yet clear due to the focus of many studies only on the p110α -interacting region of the gene. Recent data suggest that BH domain mutant forms of p85α may alter the stability of PTEN [20]. p85α binds to unphosphorylated PTEN within the high molecular weight PTEN associated complex (PAC) [22]. This interaction involves the N-terminal region of p85 (SH3-BH) and has been shown to positively regulate the lipid kinase activity of PTEN in a growth factor-dependent manner [23]. Expression of p85α with deletion of the BH domain alone significantly reduced interaction with PTEN and deletion of both domains abolished it, leading to increased amplitude and duration of AKT activation following growth factor stimulation [23]. Thus, PTEN: p85α interaction appears to potentiate the ability of PTEN to reduce PIP3 levels and terminate signaling to AKT. Indeed the mutation E160* identified in endometrial carcinoma has been shown to destabilise PTEN and result in increased AKT phosphorylation [20]. 

The BH region also shows sequence homology to RhoGAP proteins and possesses GAP activity toward Rab5, Rab4, Cdc42 and Rac1 [21]. The Rab proteins are regulators of endosome trafficking and influence vesicle fusion events during uptake, recycling or processing for degradation of growth factor receptors such as EGFR [44] and PDGFR [45]. Expression of a GAP-defective mutant of bovine p85α (R274A) [39] led to sustained levels of PDGF receptor activation and downstream MAPK signaling in response to PDGF stimulation [21]. Expression of this p85α-R274A mutant in NIH3T3 cells induced focus formation in cell monolayers, anchorage-independent growth and tumorigenicity, demonstrating that disruption of p85α GAP function can contribute to cellular transformation [39]. Thus, the SH3-BH region of p85α may be considered to exert a tumour suppressor function via positive regulation of PTEN activity or through its RabGAP activity that can impact receptor trafficking and signaling functions. Mutations that affect one or other of these functions may contribute to tumorigenesis in different ways. 

All missense mutations that we identified in this region (E137K, R262T and K288Q) are in highly conserved residues. The three point mutations and deletion identified here are remote from the region of interaction between BH domains revealed by the structure of the homodimer of amino acids 105-319 [37]. The precise residues required for Rab5 and PTEN interaction and the multiple other proteins reported to interact with p85α remain to be defined. R262 forms extensive contacts with three glutamic acid residues, one of which also interacts with K288. Mutations of R262 and/or K288, may therefore destabilise the interhelix interactions mediated by these side chains that help maintain the structure of the BH domain. The truncating mutation R162*, reported in a colorectal cancer [17], and E218* found here, could exert their effect via deletion of the more C-terminal domains involved in p110α interaction, rather than loss of BH domain function(s), though the low levels of protein expression we achieved here for E218* did not allow this to be properly assessed. 

Our results indicated that all p85 BH mutant proteins, except E218*, R162* and p85Δ, could bind p110α. As these forms do not contain the p110α-binding domain, they are not predicted to activate, regulate or stabilise p110α. Recent work that characterized an E160* mutant of p85, showed that its expression enhanced PTEN ubiquitination and interfered with PTEN and wild-type p85 binding, thus destabilising PTEN [20]. The large truncations of the p85 protein identified here (R162* and E218*) may function in a similar manner.

BH and iSH2 domain mutants of p85α show different effects on AKT activation, cell proliferation and anchorage-independent growth. p85α mutant forms that affect p110α activity normally result in activation of AKT [17,19,20]. Here, only the iSH2 domain N564D mutant induced dramatically increased AKT activation, proliferation and anchorage-independent growth. This suggests that BH domain mutants have p110α-independent functions and indicates that in contrast to i- and nSH2 mutants, the oncogenic activity of these mutants is not mediated by p110α. Further research is required to investigate the mechanisms of these BH domain mutants, particularly their effects on GAP activity and PTEN binding in urothelial cells.

In addition to mutational alterations in p85α that may interfere with protein:protein interactions, overall downregulation of the wildtype protein may contribute to tumour development. Some previous studies have suggested that levels of p85α and p110α in normal cells are tightly linked, arguing against a role for free p85α [46]. However, recent data indicate the potential importance of changes in overall levels of p85α in control of PI3K signaling and tumour development and suggest that it may exert a tumour suppressive role [23,47]. We found the majority of bladder cancer cell lines to have decreased levels of p85α expression. Reduced p85α has been shown to increase PI3K signaling in some tissues and decrease PI3K signaling in others, particularly in the context of heterozygous PTEN levels [48]. Thus, p85 has a critical role in PI3K signaling via effects on p110, but in addition it can also function in some tissues to inhibit PI3K signaling and suppress tumour formation, likely through effects on PTEN [22,49]. Overexpression of p110α has been shown to reduce p85α:PTEN heterodimers leading to the suggestion that p110α and PTEN form distinct complexes with p85α[20]. Our finding of a novel class of *PIK3R1* mutations in the region of p85α responsible for PTEN regulation in bladder cancer is consistent with this additional function for p85α. Taken together our data indicate that there are complex genomic imbalances affecting the *PIK3R1* region in bladder cancer and major alterations in expression of PIK3R1.

## Supporting Information

Figure S1 **Sanger sequencing traces showing mutations identified in exons 2-6 of *PIK3R1*.** Bracketed traces show mutations found in cis in tumor 2.
(TIF)

Figure S2 **Allele-specific PCR determination of phase of two mutations in the cell line LUCC3 and tumor sample 2.** For each pair of mutations, c.1519 G>C (exon 13) / c.1670 G>C (exon 14) in LUCC3 (A) and c.652 G>T (exon 5) / c.862 A>C (exon 6) in tumor 2 (B), primers were used to amplify a single PCR product that spanned the two mutation sites. Electrophoresis analysis shows the PCR products from different DNA templates specific for the first 5’ mutant sequence. Electropherograms show the corresponding mutant (MT) nucleotide at the alternative mutation site on the same PCR product.
(TIF)

Figure S3 **Multi-species alignment of p85 alpha protein sequence (from amino acid 81) showing conservation of residues E137, R262 and K288.**
(TIF)

Figure S4 **Anchorage-independent growth analysis of Rat1 cells expressing WT and mutant p85α.** No colonies > 50 μm were seen in vector only cells.
(TIF)

Table S1 **Urothelial carcinoma cell lines and their origins.**
(DOC)

Table S2 **Primers used for high resolution melting analysis of *PIK3R1*.**
(DOCX)

Table S3 **Allele-specific PCR to determine phase of PIK3R1 mutations.**
(DOCX)
