# Supplementary figures and images for: Identification of Mutations in Distinct Regions of p85 Alpha in Urothelial Cancer
Source: PLoS One. 2013 Dec 18;8(12):e84411. doi: 10.1371/journal.pone.0084411 (PMC3867501; doi:10.1371/journal.pone.0084411)

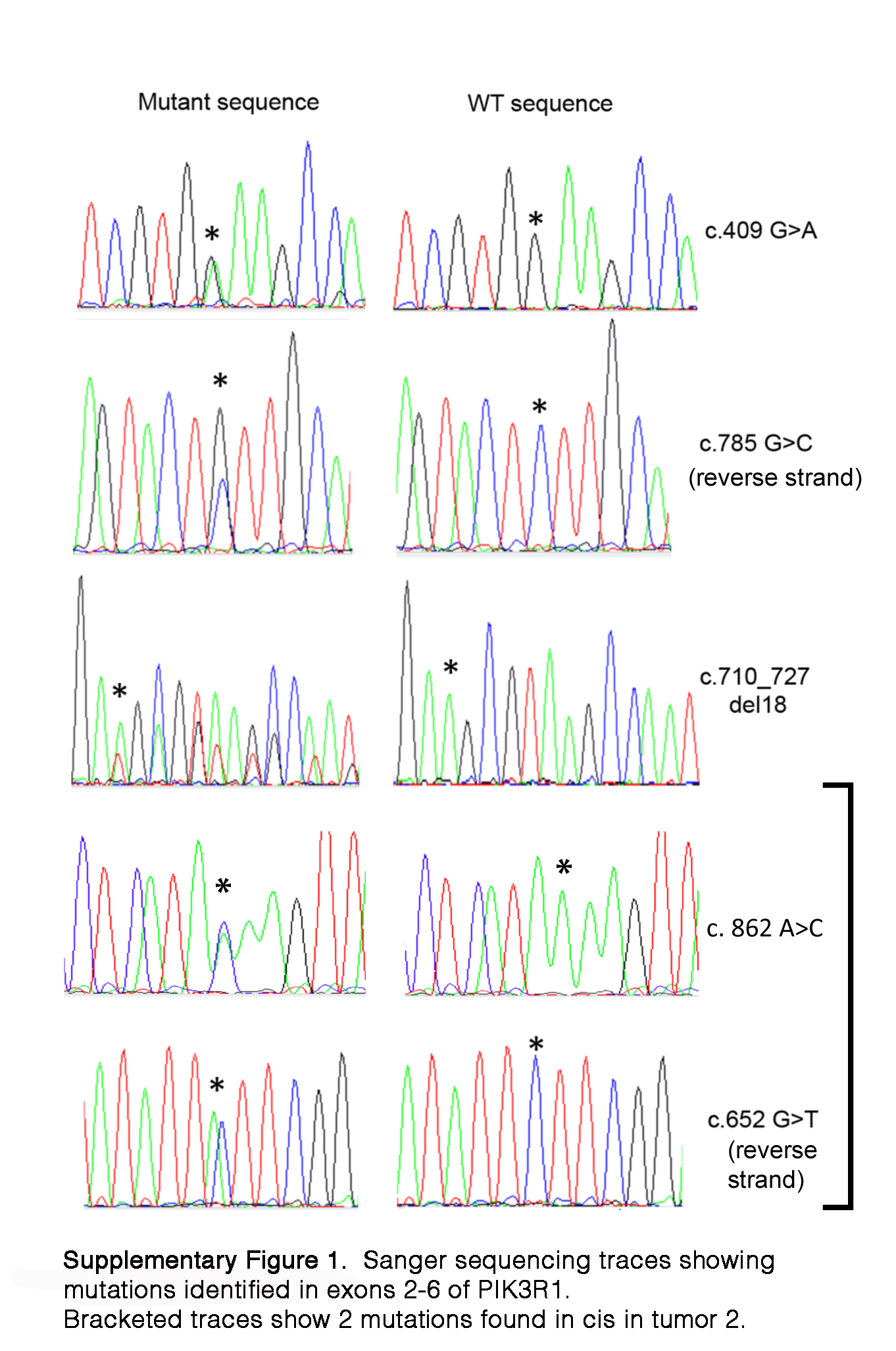

Supplement: Figure S1 — Sanger sequencing traces showing mutations identified in exons 2-6 of PIK3R1. Bracketed traces show mutations found in cis in tumor 2. (TIF) [file pone.0084411.s001.tif]

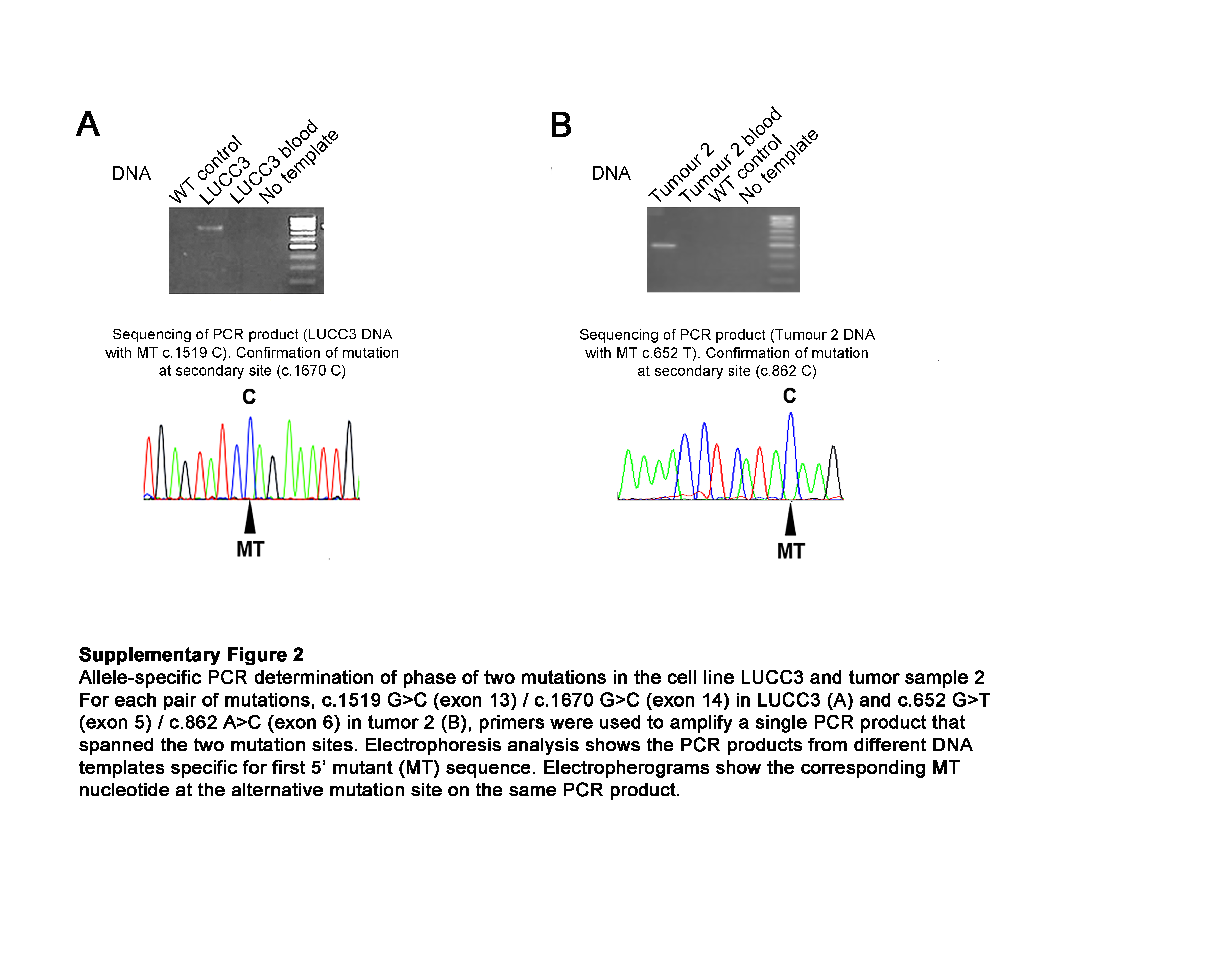

Supplement: Figure S2 — Allele-specific PCR determination of phase of two mutations in the cell line LUCC3 and tumor sample 2. For each pair of mutations, c.1519 G>C (exon 13) / c.1670 G>C (exon 14) in LUCC3 (A) and c.652 G>T (exon 5) / c.862 A>C (exon 6) in tumor 2 (B), primers were used to amplify a single PCR product that spanned the two mutation sites. Electrophoresis analysis shows the PCR products from different DNA templates specific for the first 5’ mutant sequence. Electropherograms show the corresponding mutant (MT) nucleotide at the alternative mutation site on the same PCR product. (TIF) [file pone.0084411.s002.tif]

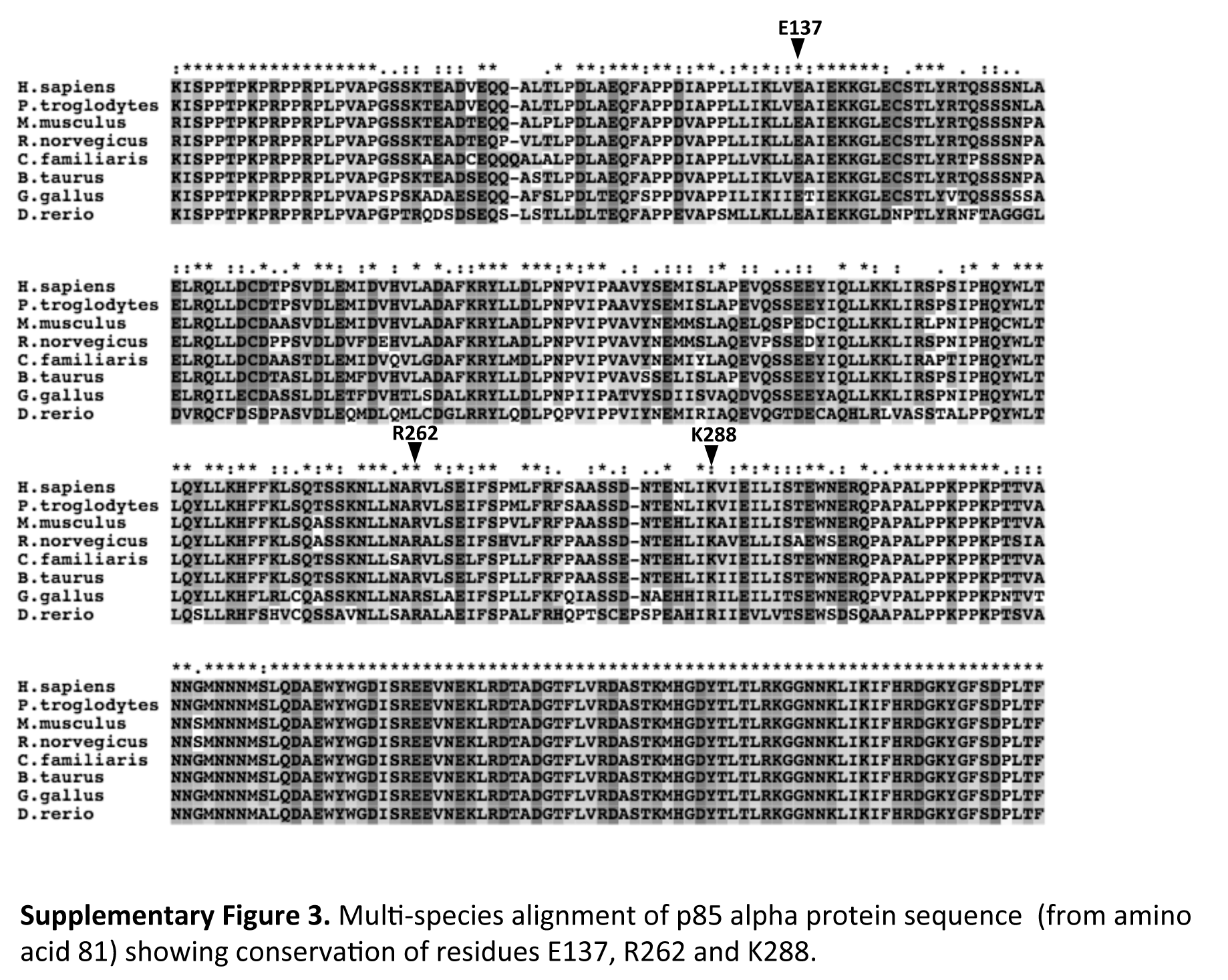

Supplement: Figure S3 — Multi-species alignment of p85 alpha protein sequence (from amino acid 81) showing conservation of residues E137, R262 and K288. (TIF) [file pone.0084411.s003.tif]

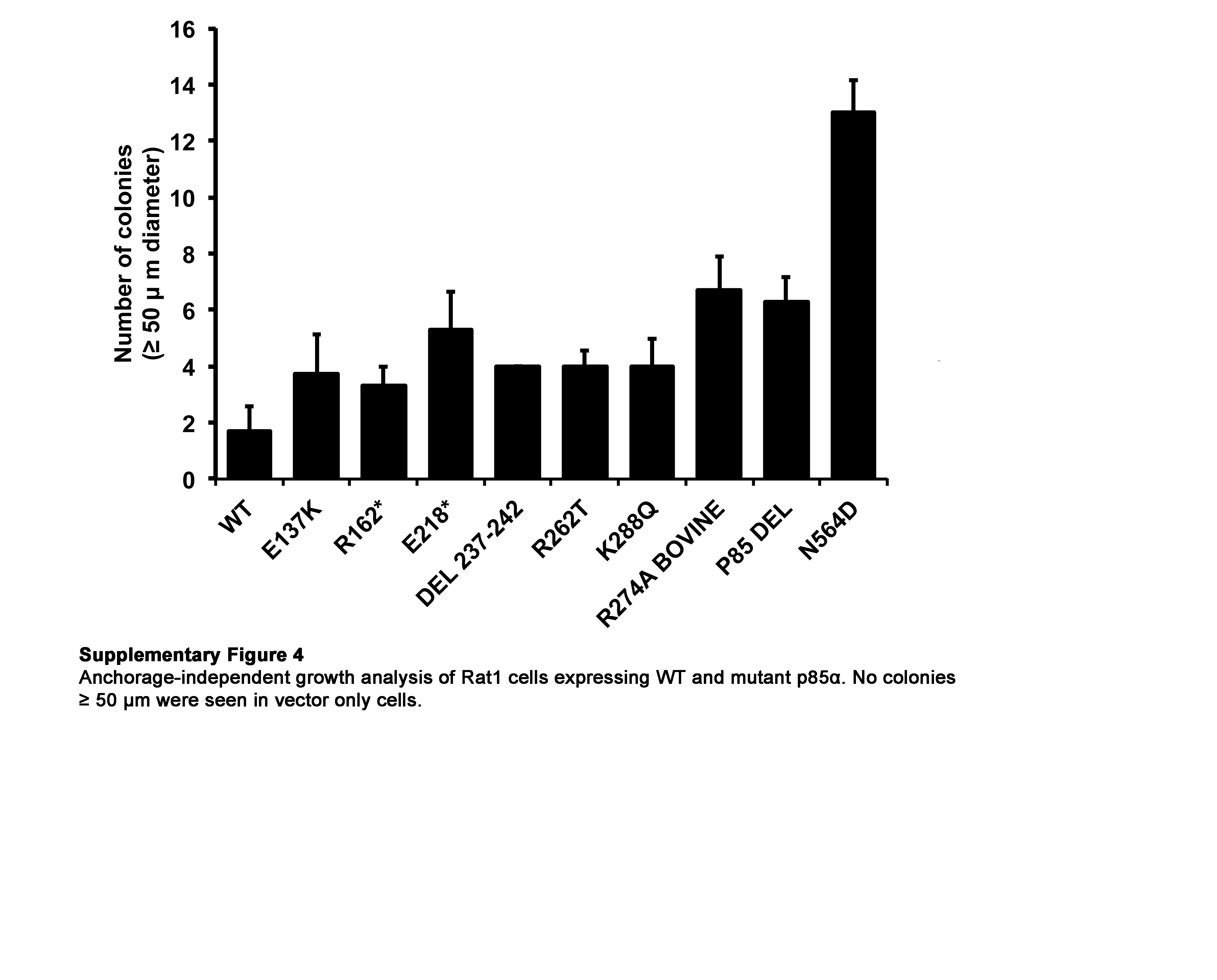

Supplement: Figure S4 — Anchorage-independent growth analysis of Rat1 cells expressing WT and mutant p85α. No colonies > 50 μm were seen in vector only cells. (TIF) [file pone.0084411.s004.tif]
