# Supplementary material for: Identification of Mutations in Distinct Regions of p85 Alpha in Urothelial Cancer
Source: PLoS One. 2013 Dec 18;8(12):e84411. doi: 10.1371/journal.pone.0084411 (PMC3867501; doi:10.1371/journal.pone.0084411)
Supplement: Table S1 — Urothelial carcinoma cell lines and their origins. (DOC) [file pone.0084411.s005.doc]

**Supplementary Table 1. Urothelial carcinoma cell lines and their origins**

| **Cell line** | **Histopathology** | **Grade** | **Stage** | **Reference** |
| --- | --- | --- | --- | --- |
| 253J | ^1^TCC | G4 | T4 |  |
| 5637 | TCC | NR | NR |  |
| 639V | TCC | G3 | NR |  |
| 647V | TCC | G2 | NR |  |
| 92-1 | TCC | G3 | T4 |  |
| 94-10 | TCC | G2/3 | T3 |  |
| 96-1 | TCC | G2/3 | T2 |  |
| 97-1 | TCC | G1/2 | T2 |  |
| 97-18 | TCC | G3 | T2 |  |
| 97-24 | TCC | G3 | T3 |  |
| 97-7 | TCC | G2/3 | T1 |  |
| BC-3C | TCC | G4 | ‘stage C’ |  |
| BFTC905 | TCC | G3 | ‘stage D1’ |  |
| BFTC909 | TCC | G3 | T4N2M1 |  |
| CAL29 | TCC | G4 | T2 |  |
| DSH1 | TCC | G2 | T1a |  |
| HT1197 | TCC | G4 | T2 (min) |  |
| HT1376 | TCC | G3 | ‘invasive’ |  |
| J82 | TCC | G3 | T3 |  |
| JMSU1 | TCC | NR | NR |  |
| JO’N | TCC | NR | NR | unpublished |
| KU-19-19 | TCC | G3 | T3b |  |
| LUCC1 | TCC | G3 | T3b | Knowles et al, unpublished |
| LUCC2 | TCC | G2 | T2a | Knowles et al, unpublished |
| LUCC3 | TCC | G3 | T2 | Knowles et al, unpublished |
| LUCC4 | TCC | G3 | T2+ | Knowles et al, unpublished |
| LUCC5 | TCC | G3 | T2+ | Knowles et al, unpublished |
| LUCC6 | TCC | G3 | Ta | Knowles et al, unpublished |
| LUCC7 | TCC | G3 | T3 | Knowles et al, unpublished |
| LUCC8 | TCC | G2 | Ta | Knowles et al, unpublished |
| MGH-U3 | TCC | G1 | ‘non-invasive’ |  |
| RT112 | TCC | G2 | NR |  |
| RT4 | TCC | G1 | T2 |  |
| SCaBER | SCC | ‘mod diff’ | ‘no metastasis’ |  |
| SD | TCC | NR | NR |  |
| SW780 | TCC | NR | NR |  |
| SW1710 | TCC | G3 | NR |  |
| T24 | TCC | G3 | NR |  |
| TCCSUP | TCC | G4 | NR |  |
| U-BLC1 | TCC | G3 | T2 at least | Bruch et al, 1999 |
| UM-UC3 | TCC | NR | NR |  |
| VM-CUB-1 | TCC | NR | NR | Williams, 1980 |
| VM-CUB-2 | TCC | NR | NR | Williams, 1980 |
| VM-CUB-3 | TCC | NR | NR | Williams, 1980 |

^1^TCC, transitional cell carcinoma; SCC, squamous cell carcinoma; NR, not recorded.

**References**

1. Elliott AY, Cleveland P, Cervenka J, Castro AE, Stein N, Hakala TR, et al. Characterization of a cell line from human transitional cell cancer of the urinary tract. J Natl Cancer Inst. 1974;53:1341-9.

2. Fogh J. Cultivation, characterization, and identification of human tumor cells with emphasis on kidney, testis, and bladder tumors. Natl Cancer Inst Monogr. 1978:5-9.

3. Elliott AY, Bronson DL, Stein N, Fraley EE. In vitro cultivation of epithelial cells derived from tumors of the human urinary tract. Cancer Res. 1976;36:365-9.

4. Elliott AY, Bronson DL, Cervenka J, Stein N, Fraley EE. Properties of cell lines established from transitional cell cancers of the human urinary tract. Cancer Res. 1977;37:1279-89.

5. Sarkar S, Julicher KP, Burger MS, Della Valle V, Larsen CJ, Yeager TR, et al. Different combinations of genetic/epigenetic alterations inactivate the p53 and pRb pathways in invasive human bladder cancers. Cancer Res. 2000;60:3862-71.

6. Pratsinis H, Saetta A, Gagos S, Davaris P. Isolation and characterization of a novel bladder cancer cell line: inhibition by epidermal growth factor. In Vitro Cell Dev Biol Anim. 1998;34:722-8.

7. Tzeng CC, Liu HS, Li C, Jin YT, Chen RM, Yang WH, et al. Characterization of two urothelium cancer cell lines derived from a blackfoot disease endemic area in Taiwan. Anticancer Res. 1996;16:1797-804.

8. Cattan N, Rochet N, Mazeau C, Zanghellini E, Mari B, Chauzy C, et al. Establishment of two new human bladder carcinoma cell lines, CAL 29 and CAL 185. Comparative study of cell scattering and epithelial to mesenchyme transition induced by growth factors. Br J Cancer. 2001;85:1412-7.

9. Williams SV, Sibley KD, Davies AM, Nishiyama H, Hornigold N, Coulter J, et al. Molecular genetic analysis of chromosome 9 candidate tumor-suppressor loci in bladder cancer cell lines. Genes Chromosomes Cancer. 2002;34:86-96.

10. Rasheed S, Gardner MB, Rongey RW, Nelson-Rees WA, Arnstein P. Human bladder carcinoma: characterization of two new tumor cell lines and search for tumor viruses. J Natl Cancer Inst. 1977;58:881-90.

11. O'Toole C, Price ZH, Ohnuki Y, Unsgaard B. Ultrastructure, karyology and immunology of a cell line originated from a human transitional-cell carcinoma. Br J Cancer. 1978;38:64-76.

12. Morita T, Shinohara N, Honma M, Tokue A. Establishment and characterization of a new cell line from human bladder cancer (JMSU1). Urol Res. 1995;23:143-9.

13. Tachibana M, Miyakawa A, Tazaki H, Nakamura K, Kubo A, Hata J, et al. Autocrine growth of transitional cell carcinoma of the bladder induced by granulocyte-colony stimulating factor. Cancer Res. 1995;55:3438-43.

14. Lin CW, Lin JC, Prout GR, Jr. Establishment and characterization of four human bladder tumor cell lines and sublines with different degrees of malignancy. Cancer Res. 1985;45:5070-9.

15. Marshall CJ, Franks LM, Carbonell AW. Markers of neoplastic transformation in epithelial cell lines derived from human carcinomas. J Natl Cancer Inst. 1977;58:1743-51.

16. Rigby CC, Franks LM. A human tissue culture cell line from a transitional cell tumour of the urinary bladder: growth, chromosome pattern and ultrastructure. Br J Cancer. 1970;24:746-54.

17. O'Toole C, Nayak S, Price Z, Gilbert WH, Waisman J. A cell line (SCABER) derived from squamous cell carcinoma of the human urinary bladder. Int J Cancer. 1976;17:707-14.

18. Paulie S, Hansson Y, Lundblad ML, Perlmann P. Lectins as probes for identification of tumor-associated antigens on urothelial and colonic carcinoma cell lines. Int J Cancer. 1983;31:297-303.

19. Williams RD. Human urologic cancer cell lines. Invest Urol. 1980;17:359-63.

20. Kyriazis AA, Kyriazis AP, McCombs WB, 3rd, Peterson WD, Jr. Morphological, biological, and biochemical characteristics of human bladder transitional cell carcinomas grown in tissue culture and in nude mice. Cancer Res. 1984;44:3997-4005.

21. Bubenik J, Baresova M, Viklicky V, Jakoubkova J, Sainerova H, Donner J. Established cell line of urinary bladder carcinoma (T24) containing tumour-specific antigen. Int J Cancer. 1973;11:765-73.

22. Nayak SK, O'Toole C, Price ZH. A cell line from an anaplastic transitional cell carcinoma of human urinary bladder. Br J Cancer. 1977;35:142-51.

23. Grossman HB, Wedemeyer G, Ren L, Wilson GN, Cox B. Improved growth of human urothelial carcinoma cell cultures. J Urol. 1986;136:953-9.
