# Supplementary material for: Identification of Mutations in Distinct Regions of p85 Alpha in Urothelial Cancer
Source: PLoS One. 2013 Dec 18;8(12):e84411. doi: 10.1371/journal.pone.0084411 (PMC3867501; doi:10.1371/journal.pone.0084411)
Supplement: Table S2 — Primers used for high resolution melting analysis of PIK3R1. (DOCX) [file pone.0084411.s006.docx]

**Supplementary Table 2. Primers used for high resolution melting analysis of *PIK3R1***

| **Exon** | **Size** | **Forward primer sequence** | **Reverse primer sequence** |
| --- | --- | --- | --- |
| 1a | 236 | caggctcaactgttgcatgg | cccctttcccctgtggttt |
| 1b | 237 | aggaagccaggcctgaagaa | agaaagggagtcattaagcaaccac |
| 2 | 198 | cgcgcgtccttcattgtggtctaatgc | ggaactgatgccctctagcagt |
| 3 | 203 | ctggaatgtctctggcagca | gacctgtctttcccatttgcat |
| 4 | 240 | tgttcagaaaattagcccaactga | tgttcaatgaggttcccagaaga |
| 5 | 294 | cctggaagtagtcgcttactcattt | ccctcatcaccccaaaatacat |
| 6 | 224 | tgagtttgcttttagggaaaaggtt | gaagctgtgttacttcaaaggaggtt |
| 7 | 209 | ggcctgtcggatacaggcatttca | gaggacagggggagtgtctc |
| 8 | 186 | gccaaggaaactcttgcaca | ggcctcgaaaaattaaaaagccacgaa |
| 9 | 205 | ttgggattgcgaacaactttt | gcaagctggtgcttttctttc |
| 10 | 167 | gatgagcattgttttgtgttttca | gcccaaaaccctaatttcagc |
| 11 | 281 | ccggtttccttattccaaaatgttaatacc | tcttgtctaaacatcgtaactgga |
| 12 | 286 | gcgctttgattaaataccttatccattgaa | ctctcacccccacctcat |
| 13 | 247 | tgtcctggtagtgtcttgcag | tctagcacaagaacaagggaaa |
| 14 | 292 | ggaccgttcctgatgtaccc | ggcctcctgaattgtagcaatcaccaa |
| 15 | 187 | ttcaggatgagttaatgcgttctc | cgcgcaaagcaagtcatgcattttcc |
| 16 | 287 | gggtatgcctagggaagaca | gcgcgttgtaaagaaaagtttgctggag |
| 17 | 289 | gcgcgaattgtcttggacaagctcaaaag | gcctcagggtggctgaact |
