# Supplementary material for: Identification of Mutations in Distinct Regions of p85 Alpha in Urothelial Cancer
Source: PLoS One. 2013 Dec 18;8(12):e84411. doi: 10.1371/journal.pone.0084411 (PMC3867501; doi:10.1371/journal.pone.0084411)
Supplement: Table S3 — Allele-specific PCR to determine phase of PIK3R1 mutations. (DOCX) [file pone.0084411.s007.docx]

**Supplementary Table 3. Allele-specific PCR to determine phase of PIK3R1 mutations**

**Primers**

| **primer name** | **primer sequence** |
| --- | --- |
| c1519F_C | ccaagagcggtacagccaa**c** |
| c1519R | tcctgaattgtagcaatcaccaa |
| c652TF | cttgtagaagtacaaagctccaaa**t** |
| c652R | attacctggtgcaggctgtc |

Underlined nucleotides : engineered mismatch to decrease primer binding stability and increase allele specificity.

Bold: wild type- or mutant-specific nucleotides for point mutations.
